# Cholera—Its Cause, Pathology and Treatment

**Published:** 1849-09

**Authors:** E. McArthur

**Affiliations:** Chicago


### ARTICLE VI.

*Cholera—Its Causes, Pathology, and Treatment.* By E. McArthur, M. D., Chicago.

The writer of this article claims no knowledge of this disease—more than what he has been able to obtain from a careful perusal of the writings of those who have had experience in its treatment, and what personal knowledge and experience, he has been able to obtain from actual attendance upon the sick, since its commencement in this city.

It began its work of death in the city of Chicago, this season, on or about the 27th day of April, and up to this time, Aug. 22d, according to the daily reports of the Board of Health, there have been 634 deaths resulting from this disease.

The general opinion of the profession, who have had experience in the development of this disease, and in its remedial treatment, is, that it is not contagious. Facts connected with its progress here, seem to justify the above conclusion.

The general causes of cholera may very properly be divided into two, Epidemic and Sporadic. According to the accounts received from India, where it first broke out, and all along in its pathway through Europe, and even in this country in 1832--33, we find it recorded, that a general cholera diathesis prevailed. This epidemic influence has prevailed to such an extent in this city, (containing a population of nearly 24,000) that for the last four or five months scarcely a family has escaped, and the individuals are scarce even, who have not been affected more or less by its depressing influence. Hence, the alarm in the community where the cholera prevails, from the fact that *all* more or less feel its enervating power.

There appears to be but little doubt, that the predisposing cause of this epidemic in some cases, and the exciting cause of it in others, are, aerial, and this not unfrequently of a local origin.

This aerial cause is often aided in its effects upon the system, by local agencies and predisposing conditions, favorable to the development of the disease. And although different persons occupying the same house, may from time to time be attacked with it, the conclusion is nevertheless irresistible, that the same morbific current and local agencies may continue to a greater or less degree, as when the first individual became affected thereby.

A German chemist, (Schonbein) claims to have recently discovered a new principle in the atmosphere, which is said to prevail in cholera times, and to which he has applied the term ozone, and which principle is described by other chemists, as being a trit-oxide of hydrogen.

It is said by some who assume to know of this principle, that cholera prevails proportionately to the quantity of it in the atmosphere.

Another view of this subject is held, to wit: "The hypothesis of insect life." The origin of the disease in the low marshy districts of Bengal, and that in seasons unusually wet, together with the oppressive heat of that climate, may seem to favor this hypothesis. Others believe it to arise from some unknown miasm, or atmospheric influence, not yet detected or analyzed, by the practical and analytical chemist.

One thing is certain, this disease developes itself most in the crowded and poorly ventillated parts of cities and large towns; in low damp places, and especially where stagnant pools and water courses abound; making it the imperative duty of the public authorities in such cities and towns, to fill up and drain such places, or cause it to be done, whenever this disease prevails, or is likely to prevail.

Small filthy buildings and apartments, and more particularly, if dark, and where the impure air is continually becoming more impure, by its being confined with little egress, and less ingress of pure air, are in an especial manner obnoxious to this pestilential disease.

Individual causes have much to do in the progress of this epidemic, such as want of wholesome food; want of proper clothing; filthiness of the body; the use of ardent spirits and other narcotics; an improper use of cathartic drugs; derangement of the digestive organs; excesses in eating and drinking, especially, in eating green fruit and vegetables; irregularity of habits, and irritability of mind; together with the influence of fear, and the depressing passions. The latter perhaps more than any other cause enumerated, tending to aggravate and hasten on the full development of the disease, when it prevails extensively.

The habitually intemperate are especially liable to attacks from cholera, and when thus attacked, fall easy and almost certain victims to its dreadful and dreaded influence. This probably is brought about as a consequence of the peculiar, irritable condition of the sero-mucous membrane of the stomach and bowels, resulting from the habitual use of alcoholic drinks. Experience thus far, during the fearful progress of the epidemic in this city, has proven this position to a full demonstration.

There appears to be much discrepancy of opinion as to the manner in which the aerial agent primarily acts upon the system, but most physicians agree as to its ultimate effects.

It will not be expected that in a short article of this kind, written for a Public Journal, that we can enter largely into the detail of the general pathology of this disease, or the physiological relations of the system as connected therewith, or even speculate much upon the varied opinions entertained by different practitioners of medicine.

We shall endeavor to be brief, and so far as possible, practical, in our deductions and conclusions. By careful observations made and repeated at the bed side, in cases of advanced cholera, it is apparent at once, that a congestive state of the internal organs, and of the venous and capillary system predominates. This congestion however, is of a passive character.

The secretions of the larger glands become entirely arrested during the progress of the disease, and at the same time a profuse serous secretion from the sanguiferous system obtains into the alimentary canal and hence, we have the distinguishing phenomena of fecal evacuation of a 'rice color,' serous vomiting, &c. In Asiatic cholera symptoms are as varied as in almost any other disease, and not unfrequently as ambiguous, requiring the utmost discernment and discrimination from the Medical attendant.

Among the premonitory symptoms of the disease, or what may with propriety be called the symptoms of the first stage, may be reckoned general lassitude, want of appetite, weakness and trembling of the extremities, flatulency of the stomach and bowels, and a general enervation of the whole system.—The tongue is sometimes covered with a white fur. This symptom was first brought to the notice of the Profession, we believe, by Prof. McNaughton, of Albany, N. Y.

A very large share of the patients from Cholera, who have fallen under our immediate notice and observation in the early stage of the disease, since its commencement in this city, have had this distinguishing pathognomic symptom. And we are informed by other physicians practicing here, that their experience corresponds in this particular.

A diarrhœa comes on in a gradual manner, sometimes, almost imperceptible in its approach, and at other times is developed in a rapid manner. With some, this diarrhœa will be attended with pain, while others, and by far the greater portion, will scarcely notice that they have any looseness of the bowels. In the commencement of this diarrhœa, the fœcal evacuations are sometimes of a billious character, and at other times they are serous; but unless the disease is checked in this incipient stage, they very soon become profuse, serous, and light colored.

In some cases marked with severity from the first onset, the stools are profuse and "rice colored" from the very commencement.

In the second stage, the diarrhœa becomes more severe, and is generally accompanied with vomiting, spasms of the extremities, of the muscles of the chest, abdomen, &c. The fœcal evacuations are more and more profuse, and "rice colored;" the vomiting greatly increases, and all the above enumerated symptoms become exceedingly aggravated.

In the stage of collapse, or what may be called the third stage of the disease, a deathly hue is manifested, which once seen, will never be forgotten by the beholder. The stage of collapse following no other disease perhaps, presents such a peculiar and distinguishing aspect.

The pulsation of the arteries of the extremities becomes almost extinct, and in many instances quite so, and that sometimes several hours before dissolution takes place. In a few instances, after the wrists had been nearly or quite pulseless for many hours, the patients have by means of proper treatment and the strong recuperative powers of nature, gradually aroused from their collapsed state, and have finally recovered. The writer has seen and professionally attended two patients, during the prevalence of the epidemic here, in [illegible]om the pulse at the wrist could not be distinguished for an hour or more, who finally recovered. A cold and clammy perspiration, and a general coldness of the whole periphery obtains, together with corrugated hands and feet, and a shrivelled appearance of the features and surface in general, which the writer has never seen result as a sequel of any other disease. The great majority of deaths resulting from the cholera, occur during this state of collapse. If the patient arouse, however, from this state, there is some hope of his final recovery. The consecutive fever which now follows as a sequence, is by some denominated the fourth stage of the disease. If the treatment has not been too stimulating during the process of the disease thus far, which we fear quite too often obtains, and local inflammation in this way induced, there will be reason to hope for a favorable termination.

Treatment.—Without reviewing the practice of any man, or set of men, in relation to the curative management of this disease, we will at once state the practice recommended and pursued by ourselves, in the treatment of the incipient, and more advanced stages of Asiatic Cholera.

And first, we conceive it to be of paramount importance to attend early to each and every symptom in the least assimilating cholera. The diarrhœa especially, which has so universally preceded the development of the disease in this place, should be at once met with its appropriate remedies. Equal parts of Paregoric Elixir, Tinct. Capsicum and Spts. Camphor, given in tea-spoon full doses to an adult, and in proportion to younger patients, and repeated every half hour or hour, as circumstances might indicate, would answer well for a domestic prescription, in the early stage of the disease. A little peppermint sling will sometimes prove effective in checking flatulency in the stomach and bowels. A tea-spoon full of Paregoric, or Bateman's Drops, repeated every hour, or at longer periods, until two or three doses had been taken, has proved sufficient to arrest the incipient diarrhœa. The following pills have been found useful in the early stage of the complaint: ℞ Opium Pulv'd, Ipecac a-a ʒss., Calomel ʒij., mix and divide into sixty pills. Dose—take one every two hours until four are taken. In most cases the above has been found sufficient to check it. Sometimes we have found it necessary after having given the above, and when a serous discharge from the bowels continued, to administer the following prescription: Take Opium pulv'd grs. vi, Camphor pulv'd, and Acetate of Lead a-a ℈j; mix and divide into 8 powders, one of which to be given once in three to six hours, according to need.

Where the alvine evacuations exhibit much billious derangement, Calomel and Rhubarb, in the proportion of x. to xii. grs. of the former, to xv. grs. of the latter, have been found useful in correcting and controlling morbid discharges.

After the progress of the disease to the second stage, or where vomiting, cramping of the stomach and muscles of the extremities, together with "rice colored" evacuations have obtained, or appear to be rapidly approaching, we have relied upon the following prescription in some fifty to sixty cases seen in this stage, which have fallen under our own immediate and personal observation—ourself and two other individuals of our own family being of the number, and have found it to meet our most sanguine expectations. To wit: ℞ Calomel. grs. xvii. to xx; Opium pulv'd, grs. ii.; Camphor, grs. v. to vii.; mix and divide into 3 powders; take one and repeat every two and a half or three hours; apply strong sinapisms to the stomach, wrists, and ancles, and if any pain or uneasy sensation in the region of the bowels, apply a large hop poultice previously steeped fifteen to twenty-five minutes in hot vinegar. And so far as we are able to judge from experience derived from the number of cases mentioned above, we are led to the conclusion that the above prescription and applications varied a little to meet particular predisposition and different constitutions, will fully meet the reasonable expectations of any practitioner of medicine.

Where there is the least appearance of collapse, it is well to apply bottles filled with hot water to the feet, knees and thighs, &c. But if a general collapse obtains, or appears to be manifesting itself, it is proper in addition to the above, to give hot brandy sling, tinct. capsicum in tea-spoon full doses in hot water, one or both every half hour or hour, and in some instanstances as often as every ten or twenty minutes; also ℞ quinine grs. vi. to x.; camphor pulv'd, grs. ii. to iv., every hour, two hours, or longer period, according to circumstances; rub the extremities and surface with hot dry flannel, with, or without powdered mustard; also with hot brandy, vinegar, spt's camphor, &c. It is of the utmost importance on the part of the Medical Attendant, not only to the patient, but to the patient's friends, and attendants generally, that he endeavor by all suitable ways and means, to enliven, and so far as it may be done with reason and propriety, to encourage the patient.

And especially is it proper for the Physician, in view of the uncertainty of human life, when this pestilential scourge is sweeping off its victims as with the besom of destruction, to warn the patient in a calm and dignified manner of his danger, so that he can if necessary make any temporal arrangement in relation to his property &c., but more especially that he may look to the Great Physician of soul and body, in this, to him, "a time of need." It is far from being the duty of any medical man, to give to his patient unqualified assurance of life, when he is evidently sinking away, and is about "to pass to that bourn from which no traveller returns." It is not expected, nor for the most part desired, that a physician should turn priest or assume the office of minister, in a full sense of those terms; but it is proper, no matter what may be his views of the Christian Religion, or whether he has any settled opinion of it or not, that he should give his patient timely warning where it is practical, so that he can, as far as may be, reflect upon approaching death, and himself act upon that which appertains to himself alone, between himself and his God.

We may seem to have digressed from our legitimate subject, although we are slow to confess it,—but we will proceed.—Where the treatment has been pursued as advised above, in the second stage, to wit: Calomel, Opium and Camphor, and collapse does did not obtain for four or five hours, we have known of no patient but that recovered, if properly attended to for a few days afterward.

